# Digital economy drives regional industrial structure upgrading: Empirical evidence from China’s comprehensive big data pilot zone policy

**DOI:** 10.1371/journal.pone.0295609

**Published:** 2023-12-08

**Authors:** Caihong Yang

**Affiliations:** 1 School of Economics, Guizhou University of Finance and Economics, Guiyang, Guizhou, China; 2 Guizhou Vocational College of Economics and Business, Duyun, Guizhou, China; Shanghai University of Electric Power, CHINA

## Abstract

With the development of the digital economy, industrial structure upgrading plays an important role in realizing high-quality development. Exploiting the quasi-natural experimental setting provided by the Big Data Comprehensive Pilot Zone (BDCPZ) policy in China in 2016, this study evaluates the impacts of the BDCPZ policies on regional industrial structure upgrading using a combination of propensity score matching and difference-in-differences (PSM-DID) with panel data of 30 regions for the period 2008–2021. The results are as follows: (1) BDCPZ policies significantly promote regional industrial structure upgrading. This finding holds after conducting the placebo test and replacing explained variables. (2) BDCPZ policies enhance upgrading through technological innovation and financial deepening. (3) Heterogeneity analysis shows that the promotional effect of BDCPZ policies on industrial structure upgrading is more obvious in economically developed regions, megacities, and east-central regions; overall, regions with high industrialization benefit more. These findings have important implications: First, they provide new empirical evidence from the perspective of policy evaluation on how the digital economy affects industrial structure upgrading. Second, this study sheds light on the mechanism underlying this relationship, helping us understand how the digital economy can further affect the development of the industrial structure. Third, the policy effect is heterogenous, providing a scientific basis for the government to formulate differentiated implementation policies for different regions. This can help local industrial transformation and upgrading, and economic development in these regions through the implementation of big data and digital technologies.

## Introduction

### Background and purpose of the study

Information technology advancements in recent years have bought major changes in social production and life. Moreover, the development of economic forms has gradually transitioned from the industrial to the digital economic era. Consequently, data has become a strategic resource for a country’s development and a key element in driving economic growth, giving rise to the economic form of the digital economy. The accelerating speed and depth of the development of the digital economy is in turn affecting competition and development patterns in the global economic system.

Conceptually, the term digital economy was first seen in the book "The Digital Economy: Promise and Peril in the Age of Networked Intelligence" published by the American scholar Don Tapscott in 1996. It describes how the Internet operates and the new economic activities that it has given rise to [1]. Subsequently, in 1998, the U.S. Department of Commerce published the study "The Emerging Digital Economy", which analyzed the key role of information technology resources on the macro- and micro-economy, increasing recognition of the concept of the digital economy [2]. Another American scholar, Beomsoo Kim, proposed a new definition for the digital economy in 2002: a special economic form in which goods and services are traded in the form of information technology in the development of society [3]. Further information technology developments and the application of big data strategies have expanded the connotations and extensions of the digital economy. Today, it is no longer limited to the use of information technology and digital technology for business activities. In the G20 Digital Economy Development and Cooperation Initiative adopted at the 2016 G20 Leaders’ Hangzhou Summit, a more authoritative definition of digital economy is provided: a series of economic activities which use digitized knowledge and information as the key factors of production, and are based on the modern information network as an important carrier, and effective use of information and communication technology (ICT) as an important driving force for efficiency improvement and optimization of the economic structure [4].

Countries around the world are actively promoting the construction and development of the digital economy. Their aim is to use digital technology and data resources to enhance the efficiency of economic operations and promote the optimization of the economic structure to catalyze changes in global factor resources and increase economic competition. The United States, as the earliest country to focus on the development of digital economy, launched the "Big Data Research and Development Initiative" in 2012 to manage and apply big data as an important national strategic resource [5], and the "The Federal Big Data Research and Development Strategic Plan" in 2016 to strengthen data research and development [6]. Initiatives in recent years include the "Digital Economy Agenda" and "A U.S. Grand Strategy for the Global Digital Economy" [7, 8]. The European Union launched the "Communication on Data-Driven Economy" strategy in 2014, advocating that European countries should seize the opportunity provided by big data development [9]. In 2021, it issued the "2030 Digital Compass: The European way for the Digital Decade" outline document, which clearly defines the vision, goals, and ways to achieve digital transformation by 2030 [10]. The United Kingdom, Japan, Australia, and other countries have also introduced policies to promote the application of big data and industrial development.

The Chinese government also attaches great importance to the development of the digital economy. The Fifth Plenary Session of the 18th Central Committee of the Communist Party of China (CPC) explicitly upgraded big data to a national strategy. Further, it launched a regional pilot program for big data development in 2016, selecting several areas for implementing comprehensive pilot zones for big data, including Guizhou, Beijing-Tianjin-Hebei, the Pearl River Delta, Shanghai, Henan, Chongqing, Shenyang, and Inner Mongolia. This policy is an important way for the Chinese government to implement its big data development strategy, directly contributing to the development of the digital economy and digital China. According to the Global Digital Economy White Paper (2023), 5 major countries including the United States, China, Germany, Japan, and South Korea have a digital economy of $31 trillion in 2022 [11]. According to the Research Report on China’s Digital Economy Development (2023), China’s digital economy reached 50.2 trillion yuan in 2022, the second largest in the world [12]. Evidently, China is committed to strengthening its big data strategy and promoting the rapid development of the digital economy.

Entering the digital economy era will certainly affect industrial development and the evolution of the industrial structure while changing the mode of economic growth. Data and technology are the main driving force of the scientific and technological revolution, which is crucial for promoting industrial structural reform and high-quality development [13]. On the one hand, the development of the digital economy includes two parts: digital industrialization and industrial digitization. Digital industrialization refers to the development of the information technology industry, including electronic information manufacturing, software and information services, the ICT industry, and other related industries. These industries are involved in value-addition to the information industry. Meanwhile, industrial digitization refers to the digital transformation of traditional industries and their upstream and downstream counterparts, realizing the integration of traditional industries with information technology. Essentially, industrial digitization is the integration part of the digital economy, or what the digital economy contributes to the primary to tertiary industries. With further integration of digital technologies and the real economy, the digital economy will promote their per capita value-added and informatization rates, thus stimulating adjustment in the industrial structure.

On the other hand, the development of the industrial structure follows a certain law: it changes from the primary to secondary industry, and then to the tertiary. Crucially, the change in per capita income causes labor mobility, which in turn leads to the law of industrial structure evolution [14]. Industrial structure upgrading is the long-term evolution and continuous dynamic change process via the adjustment of the industrial layout and development of key industries so that the different industrial factor input structures are constantly optimized to achieve resource and factor allocation efficiency. In the digital economy, data acts as a new factor in social production and life. This will require adjustments in factor allocation due to the integration of information technology with the real economy and traditional industries. Further, the new industries spawned by the digital economy will compete with traditional industries, forcing them to either reform themselves or be eliminated. The digital economy will also promote the synergistic development of different industries in the region. Together, this can lead to a change in the industrial structure. Understandably, the digital economy and development of the industrial structure are closely connected. Further, its development will inevitably influence industrial structure upgrading. The transformation and upgrading of the industrial structure is related to changes in a country’s economic structure. Moreover, it is crucial for realizing stable and sustainable economic development. Overall, an exploration of the relationship between the digital economy and industrial structure upgrading can provide valuable empirical evidence for facilitating industrial transformation and upgrading, and economic development. This study examines how the digital economy affects industrial structure upgrading, the heterogeneity in these impacts, and the underlying reasons from a multidimensional perspective.

### Literature review

Three characteristics of the rich literature on the digital economy and industrial structure upgrading are discussed below: First, different methods and dimensions are used to measure the digital economy, and analyze its role in industrial structure upgrading. Some scholars use comprehensive evaluation and multivariate statistical analyses to measure the digital economy, showing that the influence of scientific and technological innovation promotes the transformation and upgrading of industrial structure [15]. Some use the entropy method to measure the development of the digital economy, and examine the transformation and upgrading of China’s urban areas by analyzing the industrial structure using both quantitative and qualitative dimensions [16]. Barefoot et al. constructed a digital economy model based on the contribution of digital economy to GDP and analyzed its role in industrial structure upgrading [17]. Bukht and Heeks proposed definitions of digital sectors, output based on digital goods and services, and three related scopes of estimation of the digital economy [18]. Zhao et al. constructed a composite index of digital economy development based on panel data of 237 prefectural-level cities in China from the four dimensions of digital industrialization, industrial digitization, digital finance, and digital infrastructure. The study developed a comprehensive index of digital economy development to analyze the role of the digital economy in the industrial structure, revealing that the digital economy significantly promotes technological progress and human capital upgrading in Chinese cities, which in turn promotes industrial structure upgrading [19]. Using city panel data, Chang et al. empirically found that the digital economy and industrial agglomeration have significant positive rationalization and help advance the industrial structure of China’s Yangtze River Delta region [20].

Second, the impact of the digital economy on specific areas of the industry is analyzed. Some scholars analyze its impact on the green total factor productivity of the textile and garment industry, and the double mediating effect of the rationalization and advancement of industrial structure [21]. Others reveal that technological innovation has a direct mediating effect on manufacturing upgrading, and that global value chains have a positive moderating effect on the relationship between the digital economy and manufacturing upgrading [22]. Kim and Park found the key role of the ICT industry in the global network as well as the ICT industry’s facilitation of the information of other important non-ICT industries [23]. The digital economy also facilitates the development of green innovations [24]. The integration of digital technologies and green innovations plays an important role in driving and developing manufacturing, construction, and agriculture industries [25–28]. In addition, the digital economy promotes digital finance to meet the needs of consumers and enterprises in different regions, facilitates factor flows, and improves the effectiveness of resource allocation, thereby contributing to the development of new energy industries [29].

Third, the role of the digital economy and application of digital technology in economic development are analyzed in different countries. The use of information networks and digital technologies has revolutionized the elements and dynamics that drive economic growth, and are important for the high-quality development of a country’s real economy. For the United States, the development and application of ICTs has contributed to productivity recovery in the United States [30]. ICTs have significantly contributed to economic growth in the United States [31]. ICT has also significantly affected India’s economic growth [32, 33]. In China, technological change and Internet development have greatly reduced labor costs and led to industrial transformation [34]. Further, the digital economy is likely to positively affect the high-quality development of the economy by increasing entrepreneurial dynamism [35]. Clearly, ICT can significantly affect economic growth in both developed and developing countries.

Notably, the literature pays more attention to examining the digital economy’s influence on economic growth and development. The digital economy is mainly measured by constructing comprehensive indexes to evaluate the development level of the local digital economy based on different methods and dimensions. To our knowledge, few studies use China’s comprehensive Big Data pilot zones as a policy shock to study their impact on industrial structure upgrading in different regions. The implementation of the Big Data Comprehensive Pilot Zone (BDCPZ) marks the beginning of the development and layout of the big data strategy and digital economy in China. Surprisingly, few scholars have examined the effect of its implementation on industrial structure upgrading. This study intends to answer the following questions: How does the implementation of China’s BDCPZ policy (hereafter, big data policy) affect industrial structure upgrading in different regions? If so, what is the mechanism of action? Considering the heterogeneity of economic development in different regions of China, does this impact differ? How can these differences be explained?

Exploiting the quasi-natural experiment of the launch of China’s BDCPZ in 2016, we analyze the role and mechanism of the impact of China’s big data policy on regional industrial structure upgrading based on panel data of 30 provincial administrative regions in the period 2008–2021. This study makes the following contributions: First, complementing the literature, this study provides evidence on the relationship between digital economy and industrial structure upgrading using China’s big data policy as a proxy for the digital economy. Most studies use comprehensive indicators for the digital economy without fully considering the impact of big data policy shocks on the digital economy. As such, this study’s insights can be conducive for improving the relevance and effectiveness of big data policies. Second, this study reveals the mechanisms through which the digital economy affects industrial structure upgrading: technological innovation and financial deepening. This extends the analysis of the pathways of policy implementation, and helps us understand how the digital economy affects the development of the industrial structure. Crucially, it provides a basis for the government to better select and leverage the mediating effects to promote industrial structure upgrading. Third, based on the actual economic development of different regions, this study explores the differences in the impact of the digital economy on industrial structure upgrading and provides in-depth discussions from the different perspectives of the economic development level, city size, regions, and level of industrialization. This enriches relevant research and provides the government with relevant insights for formulating and implementing regionally differentiated big data policies.

The next part of the study is structured as follows: The Theoretical analysis and research hypotheses section develops the theoretical analysis and research hypotheses. The Materials and methods section outlines the materials and methods. The Empirical analysis section undertakes the empirical analysis, including robustness tests, mechanism analysis, and heterogeneity analysis. The Discussion section discusses the empirical results. Finally, The Conclusions and implications section presents the conclusions and implications of this study.

## Theoretical analysis and research hypotheses

### The direct mechanism of digital economy on industrial structure upgrading

With the development of the digital economy comes the application of digital technology, which encourages the R&D of new products by reshaping the mode of industrial R&D and breaking down information transmission barriers. Together, this can catalyze the establishment of digital platforms among enterprises and promoting resource integration, improve the efficiency of the industrial supply chain, lower the cost of products and transactions, and promote industrial structure upgrading. The digital economy affects industrial structure upgrading through the following ways:

First, the digital economy transforms traditional industries. By applying information and digital technologies, traditional industries have accelerated the speed of their digital, networked, and intelligent transformation, and are able to accurately grasp the optimal resource allocation, product production process, and market development trends; optimize the production process; customize the production plan; and digitize various types of factors, such as labor, land, capital, technology, and management. Together, this is conducive for improving resource and factor allocation, and forming new productive forces, thereby promoting the optimization and upgrading of the industrial structure. Indeed, based on industry data from the United State and United Kingdom, scholars show that ICT has positive significant effects on output growth [36]. Further, digitization is conducive for helping enterprises achieve innovation in new production processes, products, and business models [37]. By applying digital technology to the production, sales and logistics, an enterprise can improve the efficiency of various production processes; digital technology especially has a significant effect on improving green total factor productivity [38]. Meanwhile, with a digital economy, improvements in education level, employment rate, and labor productivity drive the productivity of the industry.

Second, the digital economy promotes the reorganization and differentiation of the three major industries: primary, secondary, and tertiary. The application of digital technologies, such as the Internet, artificial intelligence, blockchain, and 5G, accelerates the formation of new industries, services, and business models; builds new industrial forms; and accelerates the synergistic development between industries. Together, this promotes industrial structure upgrading. Digital industrialization promotes the new business models in the digital industry, improves the development of the service industry, and fosters new industries based on ICT, big data, software technology, and other advanced technologies [39].

Third, with the development of the digital economy has revolutionized the digital transformation of national industries and intensified market competition. Crucially, it has helped the industrial economy to spontaneously adapt to the growing market demand for services [40]. Digital applications strengthen industries ability to acquire information, overcome the barriers of monopoly, and accelerate the flow of different factors of production in different enterprises across the region. This encourages the factor flows from weakly competitive enterprises to highly competitive enterprises, and from low-efficiency industries to new high-efficiency industries. This promotes the optimization and upgrading of the industrial structure. Therefore, this study proposes the following hypothesis:

Hypothesis 1: Big data policy promotes regional industrial structure upgrading.

### The indirect mechanism of digital economy on industrial structure upgrading: Technological innovation

The implementation and promotion of big data policies have substantially increased both inputs and outputs in science and technology innovation, emphasizing the importance of innovation. To gain a foothold in international competitive markets, countries must enhance the development and application of big data, and take the road of innovation and development for promoting the development of the digital economy.

On the one hand, progress in R&D and the application of key digital technologies can enhance technological innovation capability. Big data applications can reduce enterprise costs, shorten the time-to-market, and increase the product adoption rate of customers. This is conducive to the provision of services and accelerating the innovation of new products [41, 42]. Further, the enterprise can utilize digital technology to promote innovation and entrepreneurship by changing the mechanism of value creation and value acquisition [43]. According to Schumpeter’s theory of technological innovation [44], innovation is the establishment of a new production function that continuously realizes innovation through the recombination of different production factors. Firms must take risks to innovate; however, large firms can better identify, control, and respond to risks. Schumpeter regarded innovation as the core of economic growth, and that entrepreneurship drives innovation. The author argued that the ultimate purpose of entrepreneurs to innovate is to obtain potential excess profits. As massive data resources in various industries are effectively mined and utilized, data resources, as a new factor of production, form new inputs and innovation is carried out through the continuous optimization of factor inputs. The application and circulation of data resources provide a more transparent market environment for enterprises, reduce information asymmetries as well as the risk and uncertainty of innovation and entrepreneurial activities, and effectively enhance regional innovation. Since the existence of excess profits will attract other enterprises, entrepreneurs must innovate to ensure business survival. According to the new theory of technological innovation proposed by the national innovation system school represented by Freeman and Nelson [45], the driving force of technological innovation comes not only from entrepreneurs but also from the systematic role of the state in innovation. The implementation of big data policy shows the systemic role of the state in technological innovation.

On the other hand, technological innovation can promote industrial structure upgrading. The application and promotion of digital technology accelerates the transformation and upgrading of the digitalization and intelligence of traditional industries. Further, it will create new industries. Together, this will catalyze for industrial structure upgrading. Some studies show that improving innovation ability is a key driving force for economic growth [46]. Romer’s endogenous growth model emphasizes that the intrinsic power of economic growth mainly comes from technological progress [47]. As the world’s most populous country with the largest manufacturing based and second-largest economy, China has rich production and accumulation of data resources. Consequently, the development and application of big data will inevitably bring certain opportunities for and challenges to technological innovation. Some studies show that the level of technological innovation is related to industrial structure upgrading. Technological progress is a continuous driving force for industrial structure upgrading [48, 49]. Essentially, technological innovation is the process of continuously realizing technological breakthroughs. It can promote the transformation and upgrading of all links in the industrial chain, which promotes the leapfrog upgrading of the industry [50, 51]. Simultaneously, industrial structure upgrading promotes enterprise innovation. For example, continuous innovation in energy-saving technologies can help reduce carbon emissions, and thus, build a sustainable industrial structure [52]. The government can encourage small and medium-sized enterprises to improve their innovation ability through mutual learning and communication with high-tech enterprises, helping the formed transform [53]. Therefore, this study proposes the following hypothesis:

Hypothesis 2: Big data policy promotes industrial structure upgrading by enhancing the level of technological innovation.

### The indirect mechanism of digital economy on industrial structure upgrading: Financial deepening

The big data policy will also attract the input and support of financial markets and their participants. Del et al. found that ICT helps improve bank performance and financial stability [54]. Others show that the development of finance promotes the technological innovation and development of Eurozone countries [55]. China’s document "Outline of Action for Promoting the Development of Big Data", released in 2015, clearly outlines the need to increase national financial and fiscal support for the development of the big data industry. Further, it states the need to focus on supporting the core big data technologies, construction of industrial chains and public service platforms, and demonstration of major applications, among other things. Local governments have also formulated several enterprise-friendly policies to financially support the big data industry. Guizhou Province, for example, explicitly stated promoting the application of Guizhou Province’s Big Data Comprehensive Financial Service Platform to provide financing support for small and medium-sized enterprises. Further, it proposed supporting the big data industry in Guiyang City, the administrative center of Guizhou Province, to grow bigger and stronger, and fully leveraging the role of the government funds in guiding, driving, and amplifying the development of key enterprises and construction of major projects. The local government proposed setting up a big data development fund, encouraging financial institutions to innovate financial products, and improving financial services to support the development and application of big data. Overall, through financial policies for supporting relevant big data industries, preferential policies, such as financing guarantees, financing discounts, risk investments, and compensation funds, for high-tech enterprises are being precisely formulated, financial deepening is being enhanced, and financial support for key technology industries is being strengthened. Together, this can help in realizing the optimization and upgrading of the industrial structure. Therefore, this study proposes the following hypothesis:

Hypothesis 3: Big data policy promotes industrial structure upgrading by enhancing the level of financial deepening.

## Materials and methods

### Model setting

The difference-in-differences (DID) method is a popular method in policy effect research and can effectively address the endogeneity problem. The policy dummy variable Treat is set according to whether the policy is implemented, with the experimental group assigned a value of one and the control group assigned a value of zero. The year dummy variable Year is set according to before and after the implementation of the policy, with a value of 1 assigned to the year in which the policy was implemented and subsequent years, and a value of 0 assigned to the years before the implementation. Accordingly, the sample can be divided into four groups: the control group before policy implementation (Treat = 0, Year = 0), control group after policy implementation (Treat = 0, Year = 1), experimental group before policy implementation (Treat = 1, Year = 0), and experimental group after policy implementation (Treat = 1, Year = 1). The interaction term Treat×Year of the policy dummy variable with the year dummy variable is the net effect of the policy implementation.

China officially approved the construction of a comprehensive big data pilot area in 2016. This study treats this pilot policy as a quasi-natural experiment using provincial-level data samples with the following treatment in selecting the experimental and control groups: certain pilot areas are prefecture-level cities (e.g., the Pearl River Delta and Shenyang). Since this study uses provincial panel data, the province in which the prefecture-level city is located is delineated as the experimental group. Finally, the 10 regions implementing big data policies are set as the experimental group (including Guizhou, Beijing, Shanghai, Tianjin, Hebei, Guangdong, Henan, Chongqing, Liaoning, and Inner Mongolia), and the other 20 regions not implementing policies are set as the control group.

The use of the DID method to assess policy effects requires that the experimental and control groups satisfy the parallel trend hypothesis; that is, the characteristics of all aspects of regional development in the experimental and control groups should be as consistent as possible. However, owing to the variability in the development of different countries and regions, it is difficult to satisfy this requirement. To overcome this, we utilize the Propensity Score Matching (PSM) method developed by Heckman [56], and Rosenbaum and Rubin [57]. It is based on the principle of estimating the propensity score values by selecting covariates related to the explained variable and matching them according to the propensity score values, thus eliminating sample bias. Following extant research [58], this study chooses the combined PSM-DID method to more accurately assess the impact of big data policy on regional industrial structure upgrading.

The econometric model based on the traditional DID method is set as follows:

$$Y_{it}=\alpha_{0}+\alpha_{1}{Treat}_{i}\times {Year}_{t}+\sum_{i=1}^{N}b_{j}X_{it}+\varepsilon_{it}$$ (1)

Considering PSM, the model is as follows:

$$Y_{it}^{PSM}=\alpha_{0}+\alpha_{1}{Treat}_{i}\times {Year}_{t}+\sum_{i=1}^{N}b_{j}X_{it}+\varepsilon_{it}$$ (2)

Where *Y*_*it*_ is industrial structure upgrading. *α*_0_ is a constant term. Treat is the big data policy variable assigned a value of 1 for the experimental group and 0 for the control group. *Year* is a year dummy variable assigned a value of 1 for 2016 and later years, and a value of 0 for before 2016. *α*_1_ is the coefficient of the cross-multiplier term Treat×Year, which indicates the net effect of the big data policy on industrial structure upgrading. *X*_*it*_ is the control variable that controls for variables that affect industrial structure upgrading. *ε*_*it*_ is the disturbance term.

### Data sources and processing

This study selects 30 provincial panel data of China during 2008–2021, and uses Stata software to process and estimate the data. The data are obtained from China’s Economic Prediction System (EPS) database, which is mainly derived from authoritative data released by international institutions or organizations. To ensure the validity and completeness of the data, the data are processed as follows: (1) To ensure that the characteristics of the economic systems of the sample data are as similar as possible, the sample data of Hong Kong, Macao, and Taiwan are excluded. (2) China’s Xizang region is also excluded from the sample data because of the large number of missing values in the economic data. Finally, the panel data of 30 provinces are retained as the sample, totaling 420 observations. (3) For variables with missing values, linear interpolation and extrapolation are used to supplement the missing values.

### Description of variables

#### Explained variables

The explained variable is industrial structure upgrading. It is the index of industrial structure advancement, which is mainly manifested as the process and trend of the adjustment and transformation the industrial structure of a country’s national economy from a low-level structure dominated by labor-intensive industries to a high-level structure dominated by knowledge- and technology-intensive industries. That is, it is reflected by the transition of the dominant share of output from the primary industry to the secondary and tertiary industries. Relevant studies have found that the development of information technology promotes the servitization of the economic structure as an important feature of industrial structure upgrading; servitization is measured as the ratio of the tertiary industry to the secondary industry [59]. Accordingly, this study adopts the ratio of the added value of the tertiary industry to that of the secondary industry as an indicator of industrial structure advancement or upgrading.

#### Explanatory variables

The explanatory variable is the digital economy proxy. which is the exogenous shock of big data policy implementation. The policy dummy variable is assigned a value of 1 in implemented regions; otherwise, it is 0. The time of implementation of big data policy is a time dummy variable which is assigned 1 if the year is 2016 and later; otherwise, it is 0. Finally, the cross-multiplier of the two (Treat×Year) denotes the net effect of the policy’s impact on industrial structure upgrading.

#### Control variables

Other factors related to industrial development can influence the impact of the big data policy on industrial structure upgrading. Research shows that the level of regional economic development, degree of government intervention, human capital, and population size affect the development of industrial structure [16, 60, 61]. This study considers the following control variables: (1) Regional per capita economic development level (lnpergdp), measured by the ratio of regional GDP to the total population; (2) Regional level of science and technology inputs (scitech), measured by the ratio of the fiscal science and technology expenditure to the regional fiscal expenditure; (3) Size of government (gover), measured by the ratio of government fiscal expenditure to regional GDP; (4) Education level (edulevel), measured by the ratio of education expenditure to regional GDP; (5) Regional population size (totalpo), measured by the total population of the region (10,000 people).

## Empirical analysis

### Descriptive statistical analysis

Table 1 presents the descriptive statistics of the variables. With the full sample, the mean, standard deviation, and minimum and maximum values of industrial structure upgrading (upgrade) are 1.1677, 0.6769, 0.4996, and 5.2968, respectively. The mean per capita economic development level (lnpergdp) is 10.7242, with a standard deviation of 0.5394, minimum value of 9.0852, and maximum value of 12.1226. The mean science and technology investment level (scitech) is 0.0208, with standard deviation of 0.0147, minimum value of 0.0039, and maximum value of 0.0720. These three variables exhibit large differences by region. The differences in government size and education are relatively small.

**Table 1:** Descriptive statistics.

	VarName	Obs	Mean	SD	Min	Max
Full sample	upgrade	420	1.1677	0.6769	0.4996	5.2968
	lnpergdp	420	10.7242	0.5394	9.0852	12.1226
	scitech	420	0.0208	0.0147	0.0039	0.0720
	gover	420	0.2395	0.1003	0.0874	0.6430
	edulevel	420	0.0513	0.0164	0.0248	0.0976
	totalpo	420	4.6e+03	2.8e+03	554.0000	1.3e+04
Experimental group	upgrade	140	1.3986	0.9726	0.4996	5.2968
	lnpergdp	140	10.9501	0.5746	9.0852	12.1226
	scitech	140	0.0271	0.0187	0.0054	0.0720
	gover	140	0.2102	0.0620	0.1027	0.4022
	edulevel	140	0.0457	0.0140	0.0300	0.0922
	totalpo	140	4.8e+03	3.3e+03	1.2e+03	1.3e+04
Control group	upgrade	280	1.0522	0.4203	0.5539	3.2144
	lnpergdp	280	10.6113	0.4839	9.4018	11.8280
	scitech	280	0.0176	0.0110	0.0039	0.0548
	gover	280	0.2542	0.1121	0.0874	0.6430
	edulevel	280	0.0542	0.0167	0.0248	0.0976
	totalpo	280	4.5e+03	2.5e+03	554.0000	1.0e+04

By experimental and control groups, the mean value of the index of industrial structure upgrading for the experimental and control groups are 1.3986 and 1.0522, respectively. Thus, the experimental group has more advanced industrial structure than the control group. The level of the scientific and technological input of the experimental (control) group is higher (lower) than the mean value of the whole sample. This indicates large differences in science and technology investments between different regions. This difference indicates that the experimental group attaches more importance to technological innovation than the control group.

### Empirical analysis of digital economy on industrial structure upgrading

#### Empirical analysis based on full sample DID

This section assesses the impact of big data policies on industrial structure upgrading using the DID approach. Models (1) and (2) in Table 2 report the results of the benchmark regression based on the full sample data. These models represent the results without and with control variables, respectively. The coefficients of the cross-multiplier terms DID in models (1) and (2) are 0.5735 and 0.3143, respectively, and positive at the 1% level. This suggests that the big data policy significantly upgraded the industrial structure by 57.35% and 31.43%. Further, the policy indicator for regional industrial structure upgrading is positive at the 1% level, regardless of whether control variables are added.

**Table 2:** Full sample DID and matched PSM-DID results.

Variables	Model (1)	Model (2)	Model (3)	Model (4)
	upgrade	upgrade	upgrade	upgrade
DID	0.5735***	0.3143***		
	(5.8487)	(2.9473)		
PSM-DID			0.4621***	0.1746**
			(8.5333)	(2.2746)
lnpergdp		0.4537***		0.4019***
		(4.9559)		(5.4548)
scitech		-2.2436		4.7344
		(-0.6937)		(1.4717)
gover		0.5747		0.5532
		(0.3883)		(0.4066)
edulevel		13.2347		18.4384**
		(1.4806)		(2.1488)
totalpo		-0.0001		-0.0001
		(-1.2953)		(-1.4755)
_cons	1.0857***	-3.9229***	1.0072***	-3.8489***
	(77.5079)	(-4.9726)	(196.2770)	(-5.1460)
N	420	420	496	496
Adj. R^2^	0.2576	0.5809	0.2062	0.6409
N_g	30	30	30	30

Note: t-statistics are in parentheses.

***, **, and * represent significance at the 1%, 5%, and 10% levels, respectively.

#### Empirical analysis based on PSM-DID

**Fig 1:**
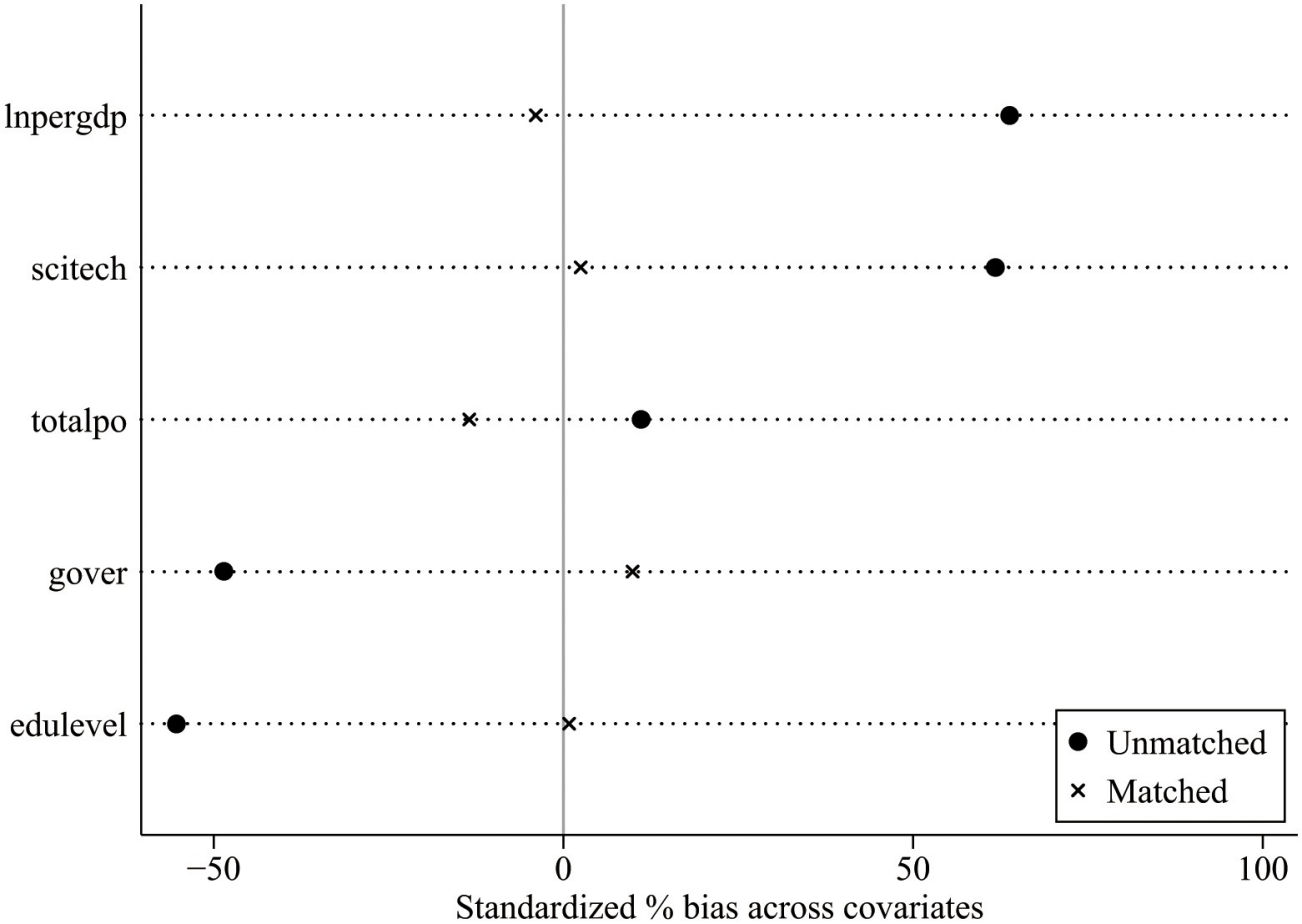
Plot of standardized deviation of covariates.

**Fig 2:**
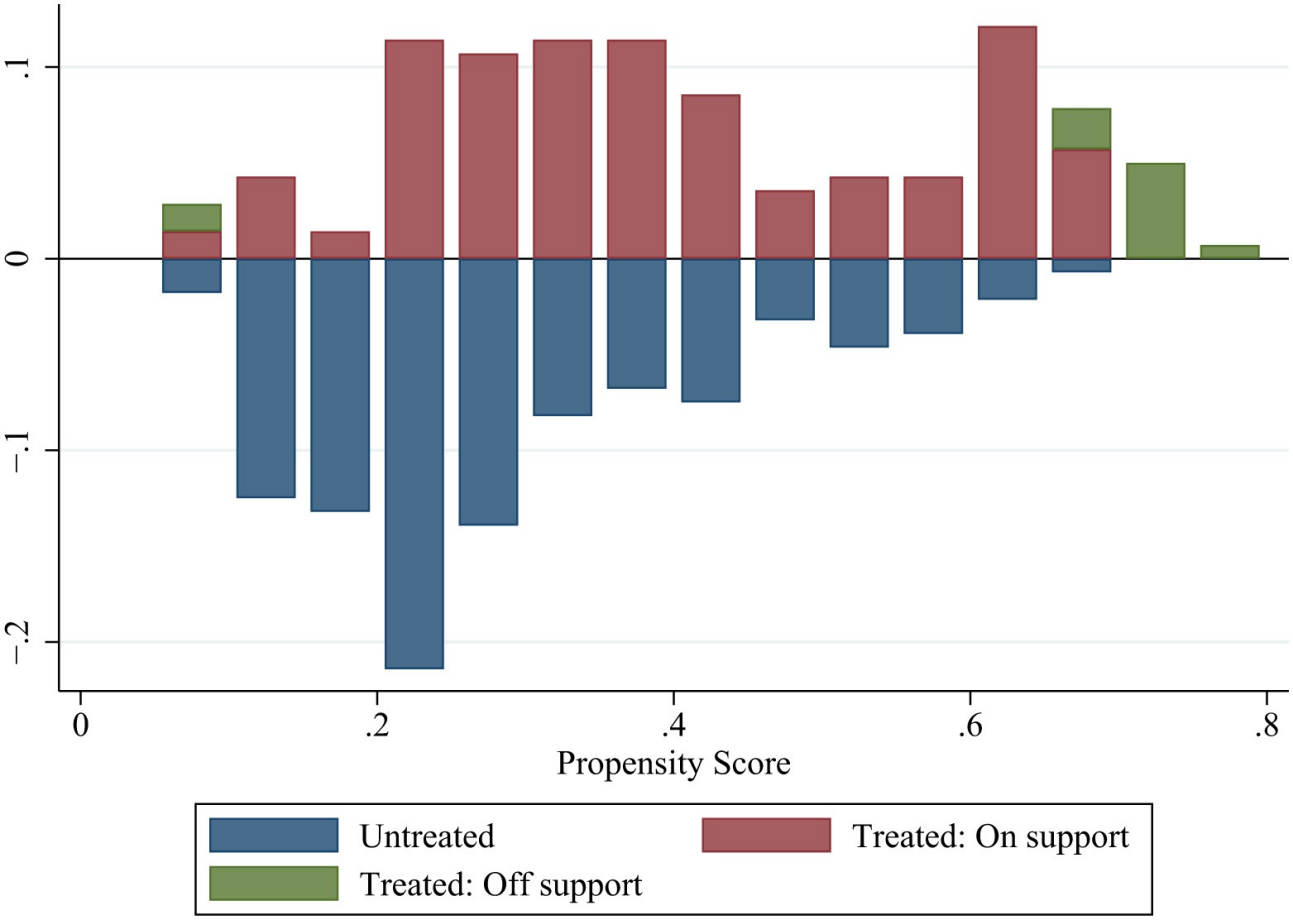
Common range of values for the propensity score.

To avoid systematic differences in the trend of changes between the experimental and control groups, and reduce sample selection bias, this study also uses the PSM-DID method. The nearest neighbor matching 1:3 criterion is used for matching. The balance test is used to check whether the matching is appropriate. Fig 1 shows the standardized deviation of the covariates after matching. The deviation between the experimental and control groups after matching is controlled as it is within 15%. Another test is the common support test. Fig 2 represents the range of common support of the sample data plotted according to the propensity score. Most sample observations are within the common range of values and the reduction in sample size during matching is limited.

Models (3) and (4) in Table 2 report the regression results after propensity score matching. The two models represent the regression results without and with control variables, respectively. The coefficients of the cross-multiplier terms PSM-DID in the models are 0.4621 and 0.1746, respectively, which are positive at 1% and 5% levels, respectively. This indicates that the big data policy significantly upgraded the level of industrial structure by 46.21% and 17.46%. The results after propensity score matching show that the big data policy still significantly promotes the regional industrial structure upgrading, regardless of whether we add the control variables. Thus, Hypothesis 1 is supported.

While the policy effect before matching is higher than that after matching, the effect remains significantly positive. This suggests that the estimation using the full sample may overestimate the impact of big data policy on regional industrial structure upgrading.

### Robustness test

#### Time placebo test: Fictitious policy time

Following relevant research [62], the placebo test of time is conducted by advancing the policy time by two to three years. Here, the placebo test serves the same function as the parallel trend test, examining the significance of the coefficients of the cross-multiplication term of the time dummy variable and the experimental group in the base regression before the occurrence of the policy. The test is passed if the coefficient is not significant. Big data was first written into the government work report in 2014 and the State Council issued the document "Outline of Action to Promote the Development of Big Data" in 2015. Hence, the dummy policy was advanced to 2012 and 2013, and included in the model with the cross-multiplier terms DID2012 and DID2013 of the experimental group. Models (5) and (6) in Table 3 report the results of the placebo test after fictionalizing the timing of the policy based on the PSM-matched data to 2012 and 2013, respectively. The estimated coefficients of the cross-multiplier terms of the different years with the experimental group before the implementation of the policy, DID2012 and DID2013, are not significant. This indicates that no significant difference between the changes in the trend of the experimental and control groups before the approval of the pilot. That is, the effect of regional industrial structure upgrading comes from the implementation of the big data policy, thereby passing the time placebo test.

**Table 3:** Placebo test results.

Variables	Model (5)	Model (6)	Model (7)	Model (8)
	upgrade	upgrade	upgrade	upgrade
DID2012	0.0137			
	(0.1975)			
DID2013		0.0745		
		(0.9813)		
DID			0.0654	0.0897
			(1.0889)	(1.6084)
lnpergdp	0.4340***	0.4173***	0.5098***	0.4254***
	(6.1991)	(5.9723)	(4.3082)	(5.4425)
scitech	3.8682	4.6122	-4.7983	2.9881
	(1.0922)	(1.3762)	(-1.0091)	(1.2026)
gover	0.3040	0.3687	0.4235	-0.5467
	(0.2387)	(0.2873)	(0.3069)	(-0.3425)
edulevel	21.9435***	20.9643**	15.1736*	22.8083*
	(2.8141)	(2.6281)	(1.7066)	(2.0019)
totalpo	-0.0001	-0.0001	-0.0000	-0.0001
	(-1.3151)	(-1.4102)	(-0.3111)	(-1.2430)
_cons	-4.4146***	-4.1559***	-4.9534***	-3.8967***
	(-6.1525)	(-5.7639)	(-4.2419)	(-4.6755)
N	496	496	420	553
Adj. R^2^	0.6171	0.6210	0.5215	0.6201
N_g	30	30	30	28

Note: t-statistics are in parentheses.

***, **, and * represent significance at the 1%, 5%, and 10% levels, respectively.

#### Cross-sectional placebo test: Fictitious experimental group

Following related research [63], fictitious experimental groups are used to conduct the cross-section placebo test. Specifically, we set up a fictitious experimental group by random sampling to examine the significance of the coefficient of the cross-multiplication term between the fictitious experimental and control groups. The test is passed if the coefficient is not significant. Because of the small number of sample cross-sections, a fictitious experimental group is set by randomly sampling once from the control group and replacing the original experimental group with the following regions: Zhejiang, Shandong, Guangxi, Sichuan, Xinjiang, Ningxia, Gansu, Hunan, Hubei, and Fujian. The other regions are set as the control group. Models (7) and (8) in Table 3 report the results before and after the propensity score matching, respectively. The estimated coefficients of the cross-multiplier term DID of the fictitious experimental and control groups are not significant before and after matching. This suggests that the policy effect of the big data policy on regional industrial structure upgrading is not influenced by other potential unobserved random factors, passing the cross-section placebo test.

#### Substitution of explained variables

Studies use different industrial structure upgrading indicators [15]. Following this, we replace our measure of industrial structure upgrading. First, we use the level of service industry (service), measured as the ratio of the added value of the tertiary industry to GDP. This can reflect how optimized is the regional industrial structure. The second indicator is the coefficient of the level of industrial structure upgrading (cyjg), measured as weighted sum of the proportion of the primary, secondary, and tertiary industries in GDP. The formula is as follows: industrial structure upgrading level coefficient = (primary industry’s share in GDP×1)+(secondary industry’s share in GDP×2)+(tertiary industry’s share in GDP×3). The larger the coefficient, the more advanced the industrial structure. If the coefficient of the cross-multiplier term DID between the experimental and control groups is still significant, then the test is passed. Models (9) and (10) in Table 4 show the policy effects on the level of service (service) before and after adding control variables. Models (11) and (12) show the policy effects on the hierarchical coefficients of industrial structural upgrading (cyjg) before and after adding control variables, respectively. The policy’s impact on alternative indicators is significantly positive after replacing the explained variables, regardless of whether control variables are added. This demonstrates the robustness of the empirical results.

**Table 4:** Results of replacing explained variables.

Variables	Model (9)	Model (10)	Model (11)	Model (12)
	service	service	cyjg	cyjg
DID	0.1050***	0.0394**	0.1234***	0.0412**
	(8.0102)	(2.4082)	(7.5384)	(2.3114)
lnpergdp		0.0969***		0.1354***
		(6.1197)		(6.9870)
scitech		1.5554*		1.6022
		(1.9168)		(1.6517)
gover		0.2400		0.4405
		(0.8450)		(1.2242)
edulevel		2.6407		1.5574
		(1.6299)		(0.8406)
totalpo		-0.0000		-0.0000
		(-0.9812)		(-0.7926)
_cons	0.4448***	-0.7301***	2.3539***	0.7619***
	(358.0404)	(-4.5079)	(1.5e+03)	(4.0082)
N	496	496	496	496
Adj. R^2^	0.2087	0.7030	0.1779	0.7064
N_g	30	30	30	30

Note: t-statistics are in parentheses.

***, **, and * represent significance at the 1%, 5%, and 10% levels, respectively.

### Mechanism analysis

In the theoretical analysis in section 2, we argue big data policy promotes industrial structure upgrading through the technological innovation and financial deepening effects. Following relevant research [64], this study adopts the stepwise test regression coefficient test to test these mechanisms. This method is divided into three steps: First, the cross-multiplier term is regressed on the explained variable. If the coefficient is significant, it indicates that the policy significantly affects the explained variable (the aforementioned empirical analysis has been verified). Second, the cross-multiplier term is regressed on the mediator variable. If the coefficient is significant, it indicates that the policy significantly affects the mediator variable. Finally, the cross-multiplier term and mediator variable are regressed on the explained variable. If the cross-multiplier term coefficient is not significant or significant with a smaller coefficient while the coefficient of the mediating variable is still significant, it indicates that the policy promotes industrial structure upgrading through the mediating variable.

#### Technological innovation effect

Technological innovation is measured by using the logarithm of the number of regional patent application acceptances (lntotalapp), which includes the number of application acceptances of the three types of patents in China: invention, utility model, and design. Models (13) to (15) in Table 5 report the results of mechanism analysis using the stepwise regression method. Models (13) and (14) indicate that policy effect on the industrial structure upgrading and technological innovation indexes are significantly positive. Model (15) indicates that after simultaneously adding the cross-multiplier term DID and technological innovation index, the significance of the DID coefficients remain unchanged, although the coefficients decrease from 0.2140 to 0.1519 while the coefficients of the technological innovation index are significant. This indicates that the policy promotes industrial structure upgrading through the technological innovation effect. Specifically, big data policy implementation can enhance technological innovation by 21.40%, whereas technological innovation can enhance industrial structure upgrading by 10.61%. Overall, big data policy enhances industrial structure upgrading by 15.19% through technological innovation effect, with the mediating effect accounting for 13% of the total effect. Thus, Hypothesis 2 is supported.

**Table 5:** Mechanism analysis results.

Variables	Model (13)	Model (14)	Model (15)	Model (16)	Model (17)
	upgrade	lntotalapp	upgrade	Find	upgrade
DID	0.1746***	0.2140***	0.1519***	0.3980***	0.0536*
	(5.5257)	(5.1392)	(4.7161)	(8.5696)	(1.7603)
lntotalapp			0.1061***		
			(3.0249)		
Find					0.3038***
					(10.6962)
lnpergdp	0.4019***	1.6365***	0.2283***	0.4576***	0.2629***
	(12.9081)	(39.8774)	(3.5042)	(9.9964)	(8.5439)
scitech	4.7344***	1.9831	4.5241**	6.7462**	2.6847
	(2.5979)	(0.8255)	(2.5025)	(2.5179)	(1.6335)
gover	0.5532	2.2560***	0.3139	5.7051***	-1.1802**
	(1.1578)	(3.5819)	(0.6537)	(8.1214)	(-2.5789)
edulevel	18.4384***	26.0246***	15.6782***	28.5251***	9.7716***
	(6.5862)	(7.0525)	(5.3669)	(6.9307)	(3.7082)
totalpo	-0.0001***	-0.0001***	-0.0001**	-0.0002***	-0.0000
	(-2.6962)	(-2.6380)	(-2.3303)	(-3.7864)	(-1.1048)
_cons	-3.8489***	-7.7269***	-3.0294***	-3.5233***	-2.7784***
	(-12.3668)	(-18.8354)	(-7.3781)	(-7.7003)	(-9.3809)
N	496	496	496	496	496
Adj. R^2^	0.6182	0.9024	0.6249	0.7258	0.6937
N_g	30	30	30	30	30

Note: t-statistics are in parentheses.

***, **, and * represent significance at the 1%, 5%, and 10% levels, respectively.

#### Financial deepening effect

The financial deepening effect is measured by the ratio of financial institutions’ deposit and loan balances to GDP (Find), where financial institutions’ deposit and loan balances are the sum of financial institutions’ balances of all deposits and loans. Models (16) and (17) in Table 5 report the results. Model (16) shows that policy effect on financial deepening is significantly positive. Model (17) shows that after simultaneously including the cross-multiplier term DID and financial deepening indicator in the regression equation, the coefficient of the cross-multiplier term DID is still significant. However, the significance and coefficient values decrease, and the coefficient of the financial deepening indicator is significant. This indicates that the policy promotes industrial structure upgrading through financial deepening. Big data policy implementation can enhance the financial deepening effect by 39.80%, and the financial deepening effect can enhance the level of industrial structure upgrading by 30.38%. Overall, big data policy enhances the level of industrial structure upgrading by 5.36% through the financial deepening effect, with the mediating effect accounting for 69.25% of the total effect. Thus, Hypothesis 3 is supported.

### Heterogeneity analysis

The implementation of big data policy clearly promotes industrial structure upgrading. Then, one may ask are the conclusions consistent for all experimental regions? Considering the regional heterogeneity in China, this study divides the sample data into groups from the following four dimensions to conduct a more in-depth analysis of the level of regional industrial structure upgrading: economic development level; city size; eastern, central, and western regions; and level of industrialization.

#### Economic development level

To consider the imbalance in China’s regional economic development, this study groups all samples according to whether the per capita GDP (pergdp) of a region in the year before the policy occurred exceeded the national per capita GDP (Chpergdp, data from China Statistical Yearbook) of that year: those whose regional GDP per capita are lower and higher than the national GDP per capita are classified as economically underdeveloped (including Guizhou, Henan, and Hebei), and economically developed regions (including Beijing, Tianjin, Shanghai, Liaoning, Guangdong, Inner Mongolia, and Chongqing, of which Chongqing is classified as an economically developed region in 2012 and later), respectively. Models (18) and (19) in Table 6 report the estimation results for economically underdeveloped and developed regions, respectively. The big data policy promotes industrial structure upgrading in economically developed regions at the 1% level, but a negative albeit insignificant effect on economically underdeveloped regions.

**Table 6:** Heterogeneity analysis (1): Economic development and city size.

Variables	Model (18)	Model (19)	Model (20)	Model (21)	Model (22)
	(Economically underdeveloped)	(Economically developed)	(Megacities)	(Megalopolis)	(Large cities)
	upgrade	upgrade	upgrade	upgrade	upgrade
DID	-0.0875	0.2668***	0.2948***	0.2058***	-0.0122
	(-1.2293)	(8.4683)	(3.7109)	(4.7421)	(-0.2049)
lnpergdp	0.4475***	0.3535***	0.5740***	0.4786***	0.2714***
	(8.4380)	(9.6158)	(5.2214)	(12.3661)	(4.7590)
scitech	10.1984***	2.5396	-2.2719	-1.2074	16.3836***
	(3.2636)	(1.3903)	(-0.4140)	(-0.6218)	(4.9556)
gover	-0.6994	1.6463**	1.4068	-0.0524	0.1429
	(-0.9750)	(2.5219)	(0.9949)	(-0.0736)	(0.1973)
edulevel	23.8992***	11.4638***	15.7403	21.7255***	25.6041***
	(5.5963)	(3.1013)	(1.3370)	(5.4292)	(6.2351)
totalpo	-0.0002	-0.0000	-0.0002*	-0.0000	-0.0002*
	(-0.9914)	(-0.9841)	(-1.7906)	(-0.5326)	(-1.7806)
_cons	-4.2209***	-3.4578***	-4.8902***	-4.9297***	-2.7046***
	(-5.4687)	(-10.2130)	(-4.3611)	(-13.9472)	(-5.1843)
N	225	271	62	232	202
Adj. R^2^	0.5693	0.7176	0.6461	0.7554	0.5908
N_g	22	14	6	10	14

Note: t-statistics are in parentheses.

***, **, and * represent significance at the 1%, 5%, and 10% levels, respectively.

#### City size

Does the effect of big data policies on industrial structural upgrading differ by city size? Indeed, the level of technological innovation and financial deepening in cities of different sizes are not the same. The impact on industrial development is also different. To analyze heterogeneity by city size, this study divides the sample into groups based on the urban population of provincial capitals: cities with an urban resident population of 10 million or more are classified as megacities (including Shanghai, Beijing, Chongqing, Guangdong, Sichuan, and Tianjin), 5–10 million are classified as megalopolis (including Hubei, Shaanxi, Zhejiang, Jiangsu, Liaoning, Shandong, Hunan, Heilongjiang, Henan, and Yunnan), and less than 5 million are classified as large cities (including Guangxi, Hebei, Shanxi, Guizhou, Anhui, Xinjiang, Fujian, Jilin, Jiangxi, Gansu, Inner Mongolia, Hainan, Qinghai, and Ningxia). Models (20)-(22) in Table 6 report the results for megacities, megalopolis, and large cities, respectively. Overall, the policy significantly promotes industrial structure upgrading more in megacities than in megalopolis, while it is negative but insignificant for large cities.

#### East, Central, and Western regions

Given regional development heterogeneities and policy priorities, the sample is grouped according to China’s division of economic regions into the eastern (Beijing, Tianjin, Hebei, Shanghai, Jiangsu, Zhejiang, Fujian, Shandong, Guangdong, Hainan, and Liaoning), central (Shanxi, Anhui, Jiangxi, Henan, Hubei, Hunan, Jilin, and Heilongjiang), and western regions (Inner Mongolia, Guangxi, Chongqing, Sichuan, Guizhou, Yunnan, Shaanxi, Gansu, Qinghai, Ningxia, and Xinjiang). To avoid the small number of samples in the northeastern region, Heilongjiang and Jilin are classified in the central region, and Liaoning is classified in the eastern region. Models (23) to (25) in Table 7 report the grouping estimation results for the eastern, central, and western regions, respectively. The big data policy promotes industrial structure upgrading in both the eastern and central regions, is significantly stronger in the central region than in the eastern region, and is not significant in the western region.

**Table 7:** Heterogeneity analysis (2): East, central, and west regions, and industrialization level.

Variables	Model (23)	Model (24)	Model (25)	Model (26)	Model (27)
	(East)	(Central)	(West)	(Low-industrialization regions)	(High-industrialization regions)
	upgrade	upgrade	upgrade	upgrade	upgrade
DID	0.2341***	0.3235***	0.0491	0.0769	0.1647***
	(5.8616)	(3.2960)	(0.8094)	(1.3014)	(4.5474)
lnpergdp	0.5403***	0.2407***	0.2895***	0.4185***	0.1488***
	(12.1101)	(3.4931)	(3.9165)	(7.8646)	(4.6606)
scitech	-0.3443	15.9054***	1.0178	-3.3994	9.1941***
	(-0.1370)	(5.5933)	(0.1658)	(-1.0469)	(5.6213)
gover	0.5923	-0.4492	0.9971	1.1301*	0.3328
	(0.6689)	(-0.3955)	(1.2625)	(1.6848)	(0.6523)
edulevel	23.6569***	27.2967***	5.7126	19.5219***	2.4976
	(6.1662)	(4.6068)	(1.1181)	(4.5281)	(0.8734)
totalpo	-0.0002***	-0.0008***	0.0008**	-0.0000	0.0001**
	(-3.4402)	(-4.8063)	(2.5444)	(-0.0746)	(2.4172)
_cons	-4.8097***	1.1720	-5.5475***	-4.3766***	-1.6517***
	(-11.9893)	(1.0177)	(-6.8899)	(-5.8522)	(-6.4002)
N	241	127	128	229	267
Adj. R^2^	0.7383	0.7361	0.5198	0.4125	0.5537
N_g	11	8	11	28	25

Note: t-statistics are in parentheses.

***, **, and * represent significance at the 1%, 5%, and 10% levels, respectively.

#### Industrialization level

The level of industrialization and industrial structure upgrading have a close connection. Specifically, when the level of industrialization of a region reaches a certain degree, urban development gains momentum. With further industrialization, the surplus labor force gradually transfers to the tertiary industry, thus realizing the upgrading of the service industry. With the fusion of information technology and industrialization, it will continuously promote economic growth and industrial structure upgrading. However, given the regional industrialization heterogeneities, this section adopts the ratio of the added value of secondary industry to regional GDP (indstr) to measure the regional industrialization level. The sample data are grouped and divided according to whether the industrialization level exceeds the median of the 30 regions (calculated based on the matched sample data, the median industrialization level is 0.4574): those below and above the median are classified as low- and high-industrialization level regions, respectively. Models (26) and (27) in Table 7 report their respective results. The coefficient of the impact of big data policy on industrial structure upgrading in high industrialization areas is 0.1647 and positive at the 1% level, whereas the impact on low industrialization areas is not significant.

## Discussion

This study analyzes the policy effects of China’s big data policy on regional industrial structure upgrading. We successively conduct empirical analysis based on the full and post-matched samples, robustness test of the empirical results, mediation analysis, and heterogeneity analysis. The findings are discussed below.

First, by evaluating the policy effect before and after propensity score matching shows that the big data policy significantly promotes regional industrial structure upgrading. Thus, the digital economy promotes regional industrial structure upgrading. This result is consistent with the literature [19, 20, 34]. However, unlike previous studies, this study analyzes the problem from the perspective of policy evaluation. The big data policy, as a means and way of digital economy development, promotes the transformation and upgrading of the industrial structure through the transformation of traditional industries and generation of new industries. The empirical results verify that the policy effects are consistent with the expected goals, affirming the positive effects of China’s construction of BDCPZ in different regions, solidify the foundation for the continued implementation and promotion of the digital strategy by government departments, and enhance the public’s confidence in and support for the implementation of the policy.

Second, mechanism analysis reveals that the digital economy promotes industrial structure upgrading through the technological innovation and financial deepening effects. Scholars generally focus on the intermediary role of technological innovation on industrial structure upgrading [15, 16, 22]. While we do find this, we also show that the financial deepening effect another mediator of the influence of the digital economy on industrial structure upgrading. Rather, this intermediary effect is more obvious than that of technological innovation. This may be because on the one hand, this study focuses on the policy shock of big data policy as a proxy variable for the digital economy. During policy implementation, the state and government behavior of the importance of the policy and capital investment. This helps highlight the financial deepening effect on industrial structure upgrading. On the other hand, scientific and technological innovation, and their transformation need a certain time. Thus, there is a certain lag in the effect of industrial structure upgrading through the improvement in technological innovation level; hence, its mediation effect is not as obvious as the financial deepening effect. Crucially, the empirical results provide a decision-making basis for the government to enhance the level of industrial structure upgrading by continuously strengthening the role of technological innovation and financial deepening.

Finally, given the heterogenous development levels and nature of development, heterogeneity analysis is carried out by grouping and dividing the sample data. The results are as follows: (1) Dividing the sample data into economically developed and economically underdeveloped regions, the results show that the digital economy significantly promotes industrial structure upgrading of the economically developed regions, albeit a negative and insignificant effect for economically underdeveloped regions. This may be because the implementation of the big data policy requires a certain economic foundation. Economically developed regions have stable and good industrial foundations, complete industrial systems, and can better utilize the policy dividend to promote industrial development, transformation, and upgrading. However, economically underdeveloped regions are faced with the problems of poor economic construction, insufficient power of industrial development, weak strength of local enterprises, and insufficient innovation ability. The initial development of the digital economy requires greater investment, which is constrained by the local economic foundation in underdeveloped regions, leading to relatively lagging and slow transformation and upgrading of industrial structure.

(2) Dividing the sample data into megacities, megalopolis, and large cities, the results show that the policy has a significantly stronger effect on promoting industrial structure upgrading in megacities than in megalopolis, whereas it is negative and not significant for large cities. This may be because compared with megalopolis and large cities, megacities have already reached a high level of economic development and industrial structure foundation. Consequently, their technological innovation ability and degree of financial deepening are very strong. This can effectively improve the advantages of urban resource allocation and efficiency; thus, the policy effect is most significant. Large cities do not have the basic conditions for industrial development because of their relatively small scale and fail to attract more factor resources; hence, they have limited competitive advantages; therefore, the policy effect is not significant.

(3) Dividing the sample data into eastern, central, and western regions, the results show that the big data policy promotes industrial structure upgrading in both the eastern and central regions, the effect in the central region is significantly stronger than in the eastern region, and it is not significant in the western region. This is consistent with the conclusions of the analysis of economically developed and underdeveloped regions. The high level of economic development and industrial foundation of the eastern and central regions allow them to fully utilize the policy dividend and regional advantages, and promote industrial structure upgrading through technological innovation and financial deepening. The policy effect in the central region is more obvious for the following reasons: On the one hand, the eastern region faces congestion problems such as resource constraints, rising costs, and high pressure on resources and the environment after decades of high development. Meanwhile, the central region has resource advantages, labor factor advantages, and market potential given its better economic construction, which has prompted the gradual transfer of industrial regions to the central region. On the other hand, in recent years, the Chinese government has issued several plans related to the development of the central region and provided corresponding policy support, such as undertaking industrial transfer, city cluster development, and other special policies.

(4) Dividing the sample data into regions with high and low industrialization levels, the results show that big data policies significantly promote industrial structure upgrading in regions with high industrialization levels, but not in low industrialization regions. This indicates that the higher the level of industrialization, the more obvious are the big data policy effects. Thus, whether a region’s industrialization level is fully developed determines whether the policy implementation can effectively enhance industrial structure upgrading. The heterogeneity analysis of previous studies is based more on the level of economic development, or by East, Central, and West regions [16]. Meanwhile, our additional analyses by city size and industrialization level enrich the literature on differences in the policy impact on the samples of different natures and the corresponding reasons. It provides finer insights for the future development of differentiated policies for the development of digital economy.

## Conclusions and implications

### Conclusion

The continued development of the digital economy has popularized and advanced the application of digital technological innovations. New industries are being spawned by leveraging these digital technologies for the continuous optimization of resource allocation. Meanwhile, traditional industries are facing changes. Together, this has promoted the continuous upgrading of and change in the industrial structure. Thus, the digital economy has become another crucial force for economic development by driving industrial upgrading. China is also promoting the digital economy through its big data policy. However, few studies have focused on the relationship between the big data policy and industrial structure upgrading in the context of the digital economy.

Exploiting the quasi-natural event of the implementation of the BDCPZ in 2016, this study uses PSM-DID to empirically analyze the impact of this big data policy on industrial structure upgrading, the underlying mechanism, and the heterogeneity of this impact differences based on the panel data of 30 regions in China for the period 2008–2021. The results show that: (1) The digital economy effectively promotes regional industrial structure upgrading, affirming the positive role of big data policies in the development of industrial structure. (2) The mechanism analysis shows that the implementation of the policy significantly promotes regional industrial structure upgrading through technological innovation and financial deepening effects. (3) Heterogeneity analysis shows that by economic development levels, the policy effect is significant for economically developed regions. By city size, both megacities and megalopolis show significant policy effects, with the policy effect being stronger for megacities than that for megalopolis. By region, the east and central regions have significant policy effects, with the policy effect being stronger in the central region than that in the eastern region. Finally, by industrialization level, the policy effect is significant in high industrialization level areas.

### Implications

Theoretically, this study first introduces big data policy as a proxy variable for the digital economy, providing a new point of departure for researching digital economy-related fields and enriching research related to the development of the digital economy and industrial structure. Second, we analyze the policy effects of China’s big data policies on industrial structure upgrading from the perspective of policy evaluation, providing a systematic, comprehensive, and scientific assessment of the implementation effects. This is helpful for discovering, summarizing, and promoting successful policy practices. This study chooses the PSM-DID method to assess the policy effects, which helps address endogeneity and sample selection bias. Finally, this study performs valuable and insightful mechanism and heterogeneity analyses on the role of the digital economy in regional industrial structure upgrading. This provides a scientific basis for finer policy design and adjustments, and can help enhance the effectiveness and relevance of policy implementation.

Practically, this study offers some policy insights. First, the application and development of big data has a positive impact and a key role in regional industrial structure upgrading. The government should vigorously develop related digital industries, and consistently promote the digital transformation of traditional industries and enterprises. Second, the government should fully leverage the supporting role of technological innovation and financial capital in industrial structure upgrading. It should encourage enterprises to innovate, strengthen their innovation capacity, improve their innovation level. Further, the government should optimize the innovation environment and provide technical support for industrial upgrading. On financial deepening, the government should focus on improving financing support and preferences for the transformation and upgrading of manufacturing, high-tech, emerging, and science- and technology-intensive industries; promote the development of the service industry; optimize the allocation of resources; and promote economic restructuring. Third, the government should comprehensively consider the heterogeneity of regional economic and social development, and formulate differentiated big data policies. For economically underdeveloped regions, large cities, and western regions, the government should strengthen the construction and coordination of industrial structure and information technology infrastructure, and introduce and cultivate enterprises with innovative capabilities. For regions with different city sizes, the government should drive the transformation and development of industrial industries with intelligent and information-based industries; enhance the industrialization level; fully leverage the advantages of resource aggregation in megacities and megalopolis; promote the development of new industries; and create a harmonious and stable environment for industrial development.

### Limitations and future research

When analyzing the heterogeneity of the impact of big data policies on industrial structure upgrading, this study probable explanation is given for the differences in policy effects. This explanation needs further verification. Further, due to the imbalance in the economic development of China’s regions and differences in the industrial environments and development levels, scholars should test whether the policy recommendations are fully applicable to other regions or industries. Future research can also refine the direction of the study, such as dividing the geographic regions. Scholars can also focus on a certain region in more detail while considering the characteristics and advantages of the economic development of different regions. The industrial fields can be divided as well, focusing on specific industries or enterprises. Meanwhile, this study examines the short-term impact of the digital economy on industrial structure upgrading, but not the long-term impact. Researchers can explore the long-term impact on industrial structure upgrading, and the dynamic change process using more comprehensive data and scientific methods.

## Supporting information

S1 File The data of variables. (DTA)

S2 File Program. (DO)

## References

[1] Tapscott D. The Digital Economy: Promise and Peril in the Age of Networked Intelligence. New York: McGraw Hill. 1996.

[2] Henry D, Cooke S, Montes S. The emerging digital economy. Washington, DC: US Department of Commerce; 1998. https://www.commerce.gov/sites/default/files/migrated/reports/emergingdig_0.pdf

[3] Kim B, Barua A, Whinston A B. Virtual field experiments for a digital economy: a new research methodology for exploring an information economy. Decision Support Systems. 2002; 32(3): 215–231. doi: 10.1016/S0167-9236(01)00094-X

[4] The G20 Digital Economy Development and Cooperation Initiative. 2016. https://www.mofa.go.jp/files/000185874.pdf

[5] Gutmann M. Big Data Research and Development Initiative. National Science Foundation; 2012. https://digitalpreservation.gov/meetings/documents/ndiipp12/Day%202/BigData_Gutmann_DP12.pdf

[6] Big Data Senior Steering Group. The Federal Big Data Research and Development Strategic Plan. The Networking and Information Technology Research and Development (NITRD); 2016. https://obamawhitehouse.archives.gov/sites/default/files/microsites/ostp/NSTC/bigdatardstrategicplan-nitrd_final-051916.pdf

[7] Alan D. Digital Economy Agenda. Washington, DC: U.S. Department of Commerce; 2016. https://www.nist.gov/system/files/documents/director/vcat/Davidson_VCAT-2-2016_post.pdf

[8] Robert DA. A U.S. Grand Strategy for the Global Digital Economy. Information Technology & Innovation Foundation (ITIF); 2021. https://itif.org/publications/2021/01/19/us-grand-strategy-global-digital-economy/

[9] Communication on data-driven economy. Brussels: European Commission; 2014. https://digital-strategy.ec.europa.eu/en/library/communication-data-driven-economy

[10] 2030 Digital Compass: the European way for the Digital Decade. Brussels: European Commission; 2021. https://eufordigital.eu/wp-content/uploads/2021/03/2030-Digital-Compass-the-European-way-for-the-Digital-Decade.pdf

[11] Global Digital Economy White Paper (2023). China Academy of Information and Communications Technology; 2023. https://www.cnr.cn/bj/chrd/20230705/t20230705_526316715.shtml

[12] Research Report on China’s Digital Economy Development (2023). China Academy of Information and Communications Technology; 2023. http://www.caict.ac.cn/kxyj/qwfb/bps/202304/P020230427572038320317.pdf

[13] Ma D, Zhu Q. Innovation in emerging economies: Research on the digital economy driving high-quality green development. Journal of Business Research. 2022; 145: 801–813. doi: 10.1016/j.jbusres.2022.03.041

[14] Clark C. The Conditions of Economic Progress. London: Macmillan; 1940.

[15] Su J, Su K, Wang S. Does the Digital Economy Promote Industrial Structural Upgrading?—A Test of Mediating Effects Based on Heterogeneous Technological Innovation. Sustainability. 2021; 13: 10105. doi: 10.3390/su131810105

[16] Guan H, Guo B, Zhang J. Study on the Impact of the Digital Economy on the Upgrading of Industrial Structures—Empirical Analysis Based on Cities in China. Sustainability. 2022; 14: 11378. doi: 10.3390/su141811378

[17] Barefoot K, Curtis D, Jolliff W, Nicholson JR, Omohundro R. Defining and Measuring the Digital Economy. BEA Working Paper. 2018. https://www.bea.gov/system/files/papers/WP2018-4.pdf

[18] Bukht R, Heeks R. Defining, Conceptualising and Measuring the Digital Economy. SSRN Electronic Journal. 2017. doi: 10.2139/ssrn.3431732

[19] Zhao S, Peng D, Wen H, Song H. Does the Digital Economy Promote Upgrading the Industrial Structure of Chinese Cities? Sustainability. 2022; 14(16), 10235. doi: doi: 10.3390/su141610235

[20] Chang K, Zhang HJ, Li BY. The Impact of Digital Economy and Industrial Agglomeration on the Changes of Industrial Structure in the Yangtze River Delta. Journal of the Knowledge Economy. 2023. doi: 10.1007/s13132-023-01448-w

[21] Zhu X, Zhang B, Yuan H. Digital economy, industrial structure upgrading and green total factor productivity—Evidence in textile and apparel industry from China. PloS ONE. 2022; 17(11): e0277259. doi: 10.1371/journal.pone.0277259 36331964 PMC9635753

[22] Li W, Li Q, Chen M, Su Y, Zhu J. Global Value Chains, Digital Economy, and Upgrading of China’s Manufacturing Industry. Sustainability. 2023; 15(10), 8003. doi: 10.3390/su15108003

[23] Kim MS, Park Y. The changing pattern of industrial technology linkage structure of Korea: Did the ICT industry play a role in the 1980s and 1990s? Technological Forecasting & Social Change. 2009; 76: 688–699. doi: 10.1016/j.techfore.2008.03.009

[24] Wen H, Zhong Q, Lee CC. Digitalization, competition strategy and corporate innovation: Evidence from Chinese manufacturing listed companies. International Review of Financial Analysis. 2022; 82: 102166. doi: 10.1016/j.irfa.2022.102166

[25] Yin S, Yu YY. An adoption-implementation framework of digital green knowledge to improve the performance of digital green innovation practices for industry 5.0. Journal of Cleaner Production. 2022; 363: 132608. doi: 10.1016/j.jclepro.2022.132608

[26] Dong T, Yin S, Zhang N. New Energy-Driven Construction Industry: Digital Green Innovation Investment Project Selection of Photovoltaic Building Materials Enterprises Using an Integrated Fuzzy Decision Approach. Systems. 2023; 11, 11. doi: 10.3390/systems11010011

[27] Dong T, Yin S, Zhang N. The Interaction Mechanism and Dynamic Evolution of Digital Green Innovation in the Integrated Green Building Supply Chain. Systems. 2023; 11, 122. doi: 10.3390/systems11030122

[28] Yin S, Wang Y, Xu J. Developing a Conceptual Partner Matching Framework for Digital Green Innovation of Agricultural High-End Equipment Manufacturing System Toward Agriculture 5.0: A Novel Niche Field Model Combined With Fuzzy VIKOR. Frontiers in Psychology. 2022; 13: 924109. doi: 10.3389/fpsyg.2022.924109 35874394 PMC9304958

[29] Wu Y, Huang S. The effects of digital finance and financial constraint on financial performance: Firm-level evidence from China’s new energy enterprises. Energy Economics. 2022; 112: 106158. doi: 10.1016/j.eneco.2022.106158

[30] Stiroh KJ. Information Technology and the U.S. Productivity Revival: What Do the Industry Data Say? American Economic Review. 2002; 92(5): 1559–1576. http://hdl.handle.net/10419/60527

[31] Oliner SD, Sichel DE. The Resurgence of Growth in the Late 1990s: Is Information Technology the Story? Journal of Economic Perspectives. 2000; 14: 3–22. doi: 10.2139/ssrn.233139

[32] Jorgenson DW, Vu KMT. The ICT revolution, world economic growth, and policy issues. Telecommunications Policy. 2016; 40(5): 383–397.

[33] Erumban AA, Das DK. Information and communication technology and economic Growth in India. Telecommunications Policy. 2016; 40(5): 412–431. doi: 10.1016/j.telpol.2015.08.006

[34] Li Y. Internet development and structural transformation: Evidence from China. Journal of Applied Finance & Banking. 2020; 10(1): 153–172. http://www.scienpress.com/Upload/JAFB%2fVol%2010_1_8.pdf

[35] Zhao T, Zhang Z, Liang S. Digital Economy, Entrepreneurship, and High-Quality Economic Development: Empirical Evidence from Urban China. Frontiers of Economics in China. 2022; 17(3): 393–426. doi: 10.3868/s060-015-022-0015-6

[36] O’Mahony M, Vecchi M. Quantifying the Impact of ICT Capital on Output Growth: A Heterogeneous Dynamic Panel Approach. Economica. 2005; 72: 615–633. doi: 10.1111/j.1468-0335.2005.0435.x

[37] Miao Z. Digital economy value chain: Concept, model structure, and mechanism. Applied Economics. 2021; 53(37): 4342–4357. doi: 10.1080/00036846.2021.1899121

[38] Liu Y, Yang Y, Li H, Zhong K. Digital Economy Development, Industrial Structure Upgrading and Green Total Factor Productivity: Empirical Evidence from China’s Cities. International Journal of Environmental Research and Public Health. 2022; 19(4): 2414. doi: 10.3390/ijerph19042414 35206606 PMC8872123

[39] Raddats C, Naik P, Bigdeli AZ. Creating value in servitization through digital service innovations. Industrial Marketing Management. 2022, 104, 1–13. doi: 10.1016/j.indmarman.2022.04.002

[40] Chen Y, Wang L. Commentary: Marketing and the sharing economy: Digital economy and emerging market challenges. Journal of Marketing. 2019; 83(5): 28–31. doi: 10.1177/0022242919868470

[41] Davenport TH. Analytics 3.0, Harvard Business Review, 2013; 91(12):64–72.

[42] Zhan YZ, Tan KH, Ji GJ, Chung L, Tseng M. A big data framework for facilitating product innovation processes. Business Process Management Journal. 2017; 23(3): 518–536. doi: 10.1108/BPMJ-11-2015-0157

[43] Bonfanti A, Giudice MD, Papa A. Italian craft firms between digital manufacturing, open innovation, and Servitization. Journal of the Knowledge Economy. 2018; 9(1): 136–149. doi: 10.1007/s13132-015-0325-9

[44] Schumpeter JA. The theory of economic development. London: Routledge; 2021. doi: 10.4324/9781003146766

[45] Freeman C. Technology Policy and Economic Performance: Lessons from Japan. London: Pinter Publishers; 1987.

[46] Feldman MP, Audretsch DB. Innovation in cities: science-based diversity, specialization and localized competition. European Economic Review. 1999; 43(2): 409–429. doi: 10.1016/S0014-2921(98)00047-6

[47] Romer PM. Increasing Returns and Long-Run Growth. Journal of Political Economy. 1986; 94: 1002–1037. doi: 10.1086/261420

[48] Ghasemaghaei M, Calic G. Assessing the impact of big data on firm innovation performance: Big data is not always better data. Journal of Business Research. 2020, 108, 147–162. doi: 10.1016/j.jbusres.2019.09.062

[49] Du K, Cheng Y, Yao X. Environmental regulation, green technology innovation, and industrial structure upgrading: The road to the green transformation of Chinese cities. Energy Economics. 2021; 98: 105247. doi: 10.1016/j.eneco.2021.105247

[50] Urbinati A, Chiaroni D, Chiesa V, Frattini F. The role of digital technologies in open innovation processes: An exploratory multiple case study analysis. R&D Management. 2020; 50(1): 136–160. doi: 10.1111/radm.12313

[51] Anderson P, Tushman ML. Technological Discontinuities and Dominant Designs: A Cyclical Model of Technological Change. Administrative Science Quarterly. 1990; 35(4): 604–633. http://www.jstor.org/stable/2393511

[52] Cheng Y, Awan U, Ahmad S, Tan Z. How do technological innovation and fiscal decentralization affect the environment? A story of the fourth industrial revolution and sustainable growth. Technological Forecasting and Social Change. 2021; 162: 120398. doi: 10.1016/j.techfore.2020.120398

[53] Lahorgue MA, Cunha ND. Introduction of innovations in the industrial structure of a developing region: The case of the Porto Alegre Technopole ‘Home Brokers’ Project. International Journal of Technology Management & Sustainable Development. 2004; 2(3): 191–204. doi: 10.1386/ijis.2.3.191/1

[54] Del Gaudio BL, Porzio C, Sampagnaro G, Verdoliva V. How do mobile, internet and ICT diffusion affect the banking industry? An empirical analysis. European Management Journal. 2021; 39(3): 327–332. doi: 10.1016/j.emj.2020.07.003

[55] Pradhan RP, Arvin MB, Hall JH. Nair M. Innovation, financial development and economic growth in Eurozone countries. Applied economics letters. 2016; 23(16): 1141–1144. doi: 10.1080/13504851.2016.1139668

[56] Heckman J. The Common Structure of Statistical Models of Truncation, Sample Selection, and Limited Development Variables and a Simple Estimation for Such Models. Annals of Economic & Social Measurement.1976; 5(4): 475–492. http://www.nber.org/chapters/c10491

[57] Rosenbaum PR, Rubin DB. The Central Role of the Propensity Score in Observational Studies for Causal Effects. Biometrika. 1983; 70(01): 41–55. doi: 10.1093/biomet/70.1.41

[58] Shi DQ, Ding H, Wei P, Liu JJ. Can Smart City Construction Reduce Environmental Pollution. China Industrial Economics. 2018; (06): 117–135. doi: 10.19581/j.cnki.ciejournal.2018.06.008

[59] Gan CH, Zheng RG, Yu DF. An Empirical Study on the Effects of Industrial Structure on Economic Growth and Fluctuations in China. Economic Research Journal. 2011; 46(05): 4–16+31.

[60] Yuan H, Zhu CL. Do National High-Tech Zones Promote the Transformation and Upgrading of China’s Industrial Structure. China Industrial Economics. 2018; (08): 60–77. doi: 10.19581/j.cnki.ciejournal.2018.08.004

[61] Liang YF, You SY, Liu BX. Population Agglomeration, Human Resources Matching and Industrial Upgrading. Northwest Population Journal. 2023; 44(05): 84–97.

[62] Liu RM, Zhao RJ. Has the national high-tech zone promoted regional economic development?—Verification based on double difference method. Journal of Management World. 2015; (08): 30–38.

[63] Yu DS, Li XP, Li H. Can the Belt and Road Initiative Reduce Pollution in Cities? -Evidence from Quasi-natural Experiments. Statistical Research. 2021; 38(06): 44–56. doi: 10.19343/j.cnki.11-1302/c.2021.06.004

[64] Baron RM, Kenny DA. The moderator-mediator variable distinction in social psychological research: Conceptual, strategic, and statistical considerations. Journal of Personality and Social Psychology. 1986; 51: 1173–1182. doi: 10.1037//0022-3514.51.6.1173 3806354

